# Considerations for future studies on the effect of late phase oxygen strategies on retinopathy of prematurity

**DOI:** 10.3389/fped.2022.975613

**Published:** 2022-10-24

**Authors:** Charlotte M. Nusman, Nicoline E. Schalij-Delfos, Rolf H. H. Groenwold, Wes Onland

**Affiliations:** ^1^ Department of Neonatology, Emma Children's Hospital, Amsterdam University Medical Centers, Amsterdam, Netherlands; ^2^ Department of Ophthalmology, Leiden University Medical Center, Leiden, Netherlands; ^3^ Department of Clinical Epidemiology, Leiden University Medical Center, Leiden, Netherlands; ^4^ Department of Biomedical Data Sciences, Leiden University Medical Center, Leiden, Netherlands

**Keywords:** retinopathy, prematurity, oxygen, hemoglobin, saturation, study design

## Abstract

Strategies to ensure high intraocular oxygen delivery to the developing retina after 32 weeks gestational age, such as higher saturation targets and/or higher hemoglobin levels, are hypothesized to prevent ophthalmological treatment for retinopathy of prematurity (ROP). This short report summarizes the current evidence of these strategies, and discusses possibilities of future studies. A large sample size would be required and therefore the feasibility of a future randomized controlled trial is questioned.

## Introduction

ROP is a disorder of the developing retina in preterm infants, potentially leading to blindness or severe visual impairment (1). The pathophysiology of ROP can be divided in two phases after birth, with the turning point around 32 weeks postmenstrual age, independent of postnatal age. The early phase is characterized by relative hyperoxia and leads through suppression of vascular endothelial growth factor (VGEF) to a stop in vessel growth and vaso-obliteration. The late phase concerns hypoxia leading to formation of new vessels through production of VGEF, which can be excessive and consequently cause of problematic retinal neovascularization (2). Oxygen is considered the key player in ROP pathophysiology, but it is important to be aware of other risk factors for ROP as potential targets of interventions, such as poor nutrition status, low insulin-like growth factor 1, hyperglycemia and neonatal infections (2).

Two major therapeutic strategies aiming to influence availability of oxygen in both phases include saturation- and hemoglobin levels. As the intended amount of oxygen to prevent ROP in the two pathophysiologic phases is opposite (i.e., hypoxia in the early phase and hyperoxia in the late phase), separate evaluation of therapeutic strategies in each phase is necessary to prevent neutralizing results. Although theoretically these two phases have a gradual overlap around 32 weeks postmenstrual age, pragmatically a distinction between early and late phase is warranted by the fact that oxygen-related late phase interventions are performed in the weeks before ROP treatment, which is on average at 37 weeks postmenstrual age (3). This short report focusses on late phase oxygen strategies and describes opportunities and difficulties when designing a study on the effect of late phase oxygen strategies in preterm infants to prevent development and treatment for ROP.

## Discussion

### Current evidence

A recent review summarized late phase oxygen strategies, but did not include the targeting of hemoglobin levels, which will be discussed below (4).

Two studies provided evidence on the beneficial effect of supplemental oxygen in the late phase of ROP prevention, one randomized controlled trial (RCT) and one observational cohort study (5, 6). The STOP-ROP trial (*n* = 649) randomized patients with prethreshold ROP into a conventional oxygen arm (89%–94%) and a supplemental oxygen arm (96%–99%). Results showed no differences between these two groups in terms of progression to treatment. However, this might be due to a lack of statistical power, since the planned sample size in this study was not achieved. Interestingly, a post-hoc subgroup analysis of infants without plus disease (i.e., proliferation of vessels, similar to stage 3 ROP) did show a significant difference of progression to treatment in favor of higher saturation targets (46% vs. 32%, *p* = 0.004) (5). A retrospective cohort study (*n* = 225) compared a stable saturation target range of 90%–95% from 32 weeks PMA in one epoch (2002–2007) to an increased saturation target of >94% when infants developed prethreshold ROP in a second epoch (2008–2012). The latter strategy significantly decreased the risk of ROP progression from 44% to 23% (6).

Evidence on the effect of hemoglobin levels on retinopathy of prematurity is widely available, e.g., recently two large RCTs (*n* = 1,824 and *n* = 1013) showed no effect of transfusion thresholds on retinopathy of prematurity (7, 8). However, these studies cover both pathophysiologic phases of ROP and therefore no conclusions can be drawn on the hypothetical positive effect of higher hemoglobin levels in the late phase. Maintaining higher hemoglobin levels selectively in the late phase is investigated in one small RCT (*n* = 50). Preterm infants with birthweight <1,250 grams were randomized from 29 days postnatal age to high (40%) or low (20%–30%, depending on symptoms) hematocrit levels as threshold for erythrocyte transfusion. No differences were found between the two groups in terms of incidence and severity of ROP. This RCT has several important limitations, including its limited sample size and overlap between early and late phase of ROP development (9).

When considering efficacy of these late phase oxygen strategies, potential side effects need to be considered as well. Higher supplemental oxygen use in the late phase of ROP prevention might lead to a higher risk of adverse pulmonary events such as bronchopulmonary dysplasia and pneumonia, and increased duration of hospitalization at 3 months of corrected age as shown by the STOP-ROP trial. In contrast with these adverse findings, recent evidence shows that a liberal threshold (i.e., more erythrocyte transfusions) might not have similar side effects as supplemental oxygen (7, 8).

### Considerations for a randomized controlled trial

Despite the fact that there is considerable evidence on oxygen strategies for the prevention of ROP, a gap of knowledge remains on optimal treatment of late phase development of ROP. The relevant research question for a future study would be: what is the safety and efficacy of two interventions aiming for higher intraocular oxygen availability, i.e., high targeted saturation and high hemoglobin levels, on the development of severe ROP in the late phase?

The preferred strategy of inclusion criteria avoiding unnecessary exposure to high oxygen levels to all preterm infants is including those preterm infants with a progression towards treatment requiring ROP, e.g., stage 2 or higher.

The interventions comprise higher targeted saturation and high hemoglobin levels. Standard saturation targets are 90%–95% with supplemental oxygen and 90%–100% without supplemental oxygen; suggested interventional target is a saturation above 94% - with or without supplemental oxygen (4). Standard cut-off values of hemoglobin levels for erythrocyte transfusion are 7.3–8.9 g/dl (depending on respiratory conditions and growth) and for example 11.3 g/dl in the intervention group.

A suggested study design would be a 2 × 2 factorial design, in which patients receive either none of the interventions, one of the interventions, or both interventions. This study design enables simultaneous assessment of two interventions, requiring fewer patients than two separate two-arm parallel group RCTs. Furthermore, it allows assessment of separate as well as combined effects of the interventions (10).

The primary outcome measure could be the number of ophthalmological treatments (laser therapy or anti-VEGF). Obviously, long term outcomes such as visual impairment and blindness are crucial at study follow-up. Secondary outcome measures would include mortality, hospitalization, sepsis, bronchopulmonary dysplasia.

The sample size calculation of the 2 × 2 factorial design is based on the intervention warranting the largest sample, because this will be sufficient also for the other intervention in need of a smaller sample size (11). The effect size of adjusted hemoglobin levels is not available from the literature. However, well-conducted pilot studies are available to assist sample size calculations for future definitive trials. In absence of available evidence on the intervention of adjusted hemoglobin levels, the estimated absolute risk reduction of adjusted saturation targets was the pragmatic and estimated starting point for the 2 × 2 factorial design. Table 1 presents five scenarios of sample size calculations, as performed with the nQuery software. An estimation of the effect size or absolute risk reduction of high targeted saturation can be extracted from either the aforementioned STOP-ROP study or the retrospective study from Colaizy et al. and varies between 7% and 21% (5, 6). The percentage of patients warranting treatment in the different groups (either high targeted saturation or standard saturation) was 48% vs. 41% in STOP-ROP scenario 1, 46% vs. 32% in STOP-ROP scenario 2 (subgroup analysis concerning patients with prethreshold ROP) and 44% vs. 23% in Colaizy et al. scenario 3 (5, 6). When taking the average absolute risk reduction of these three scenarios (7 + 14 + 21 = 42, divided by 3 equals 14%), the 48% in the standard treatment arm is expected to reduce ROP stage 2 or higher by an absolute 14% (scenario 4), leading to a sample size of 193 preterm infants with ROP stage 2 or higher per group. Numerous procedures to correct for multiple testing of the separate effects of both treatments are available (12); below an example with the Bonferroni method is proved. This comprises a more stringent Bonferroni-corrected, significance level (*α* = 0.025) (scenario 5). Ultimately, one should preferably aim for a power of 90%, as these trials are difficult to perform and unlikely to be repeated, leading to a number of 305 patients per group (scenario 6). Investigating the interactions between the two intervention is not expected to be an explicit aim of the future study, partly because this will further inflate the sample size (11).

**Table 1:** Different scenarios for sample size calculations for a RCT investigating the effect of oxygen strategies on the progression to ophthalmologic treatment for ROP^a^.

Scenario	1	2	3	4	5	6
References	(5)	(5)	(6)	(5, 6)	(5, 6)	(5, 6)
Significance level (*α*)	0.05	0.05	0.05	0.05	0.025	0.025
Group 1 proportion	0.480	0.460	0.440	0.480	0.480	0.480
Group 2 proportion	0.410	0.320	0.230	0.340	0.340	0.340
Odds ratio	0.753	0.552	0.380	0.534	0.534	0.534
Power (%)	80	80	80	80	80	90
Sample size (*n*) per group	791	190	79	193	234	305

^a^ all statistical tests performed for this table are 2-sided.

Based on a nationwide study in the Netherlands, approximately 40 treatments per year are performed (average over the years 2013–2016, both laser and anti-VEGF) (3). Based on the STOP-ROP study, progression from ROP stage 2 to treatment is 48% when receiving standard saturation targets (5). Therefore, the total number of eligible patients with ROP stage 2 per year is 40/48 × 100 = 83. The total number of 610 patients for the 2 × 2 factorial design (scenario 6) would require an inclusion period of nearly 9.5 years, taking an informed consent refusal rate of 30% into account.

The high required number in patients is mainly caused by a relative small expected effect size combined with low incidence of patients with ROP stage 2. This forms a big hurdle for practical implementation of the RCT, e.g., with multinational initiatives. The question that rises is whether the effort of such a comprehensive study is in proportion to the expected benefit for the patient group.

## Conclusion

Two strategies aiming to increase oxygen availability to the retinal tissue in the late phase of ROP development are hypothesized to decrease the rate of ophthalmological treatment in patients with ROP stage 2. Assertion of higher saturation targets and higher hemoglobin levels have been insufficiently studied. A preferred study design to assess the effect of these two interventions simultaneously would be a 2 × 2 factorial design RCT. However, the high required number of patients to perform this study, forms a major drawback. Therefore, future studies investigating this topic should aim for either multinational initiatives or second-best alternatives such as a prospective observational study or retrospective studies comparing different centers or timeframes.

## Data Availability

The original contributions presented in the study are included in the article/Supplementary Material, further inquiries can be directed to the corresponding author/s.

## References

[1] Blencowe H Lawn JE Vazquez T Fielder A Gilbert C. Preterm-associated visual impairment and estimates of retinopathy of prematurity at regional and global levels for 2010. Pediatr Res. (2013) 74(Suppl 1):35–49. 10.1038/pr.2013.205 24366462 PMC3873709

[2] Hellstrom A Smith LE Dammann O. Retinopathy of prematurity. Lancet. (2013) 382:1445–57. 10.1016/S0140-6736(13)60178-6 23782686 PMC4389630

[3] Trzcionkowska K Vehmeijer W Kerkhoff FT Bauer NJC Bennebroek CAM Dijk PH Increase in treatment of retinopathy of prematurity in The Netherlands from 2010 to 2017. Acta Ophthalmol. (2021) 99:97–103. 10.1111/aos.14501 32701185 PMC7891652

[4] Raghuveer TS Zackula R. Strategies to prevent severe retinopathy of prematurity: a 2020 update and meta-analysis. Neoreviews. (2020) 21:e249–e63. 10.1542/neo.21-4-e249 32238487

[5] Flynn JT Bancalari E. On “supplemental therapeutic oxygen for prethreshold retinopathy of prematurity (stop-rop), a randomized, controlled trial. I: primary outcomes”. J Am Assoc Pediatr Ophthalmol Strabismus. (2000) 4:65–6. 10.1067/mpa.2000.105823 10773803

[6] Colaizy TT Longmuir S Gertsch K Abramoff MD Klein JM. Use of a supplemental oxygen protocol to suppress progression of retinopathy of prematurity. Invest Ophthalmol Vis Sci. (2017) 58:887–91. 10.1167/iovs.16-20822 28159975

[7] Franz AR Engel C Bassler D Rudiger M Thome UH Maier RF Effects of liberal vs restrictive transfusion thresholds on survival and neurocognitive outcomes in extremely low-birth-weight infants: the ettno randomized clinical trial. JAMA. (2020) 324:560–70. 10.1001/jama.2020.10690 32780138 PMC7420159

[8] Kirpalani H Bell EF Hintz SR Tan S Schmidt B Chaudhary AS Higher or lower hemoglobin transfusion thresholds for preterm infants. N Engl J Med. (2020) 383:2639–51. 10.1056/NEJMoa2020248 33382931 PMC8487591

[9] Brooks SE Marcus DM Gillis D Pirie E Johnson MH Bhatia J. The effect of blood transfusion protocol on retinopathy of prematurity: a prospective, randomized study. Pediatrics. (1999) 104:514–8. 10.1542/peds.104.3.514 10469778

[10] Montgomery AA Peters TJ Design LP. Analysis and presentation of factorial randomised controlled trials. BMC Med Res Methodol. (2003) 3:26. 10.1186/1471-2288-3-26 14633287 PMC305359

[11] Brookes ST Whitely E Egger M Smith GD Mulheran PA Peters TJ. Subgroup analyses in randomized trials: risks of subgroup-specific analyses; power and sample size for the interaction test. J Clin Epidemiol. (2004) 57:229–36. 10.1016/j.jclinepi.2003.08.009 15066682

[12] Vickerstaff V Omar RZ Ambler G. Methods to adjust for multiple comparisons in the analysis and sample size calculation of randomised controlled trials with multiple primary outcomes. BMC Med Res Methodol. (2019) 19:129. 10.1186/s12874-019-0754-4 31226934 PMC6588937

